# The Conservative Treatment of Injuries of the Extremities1Read before the Section on Surgery, Buffalo Academy of Medicine, February 5, 1901.

**Published:** 1901-06

**Authors:** Chauncey Pelton Smith

**Affiliations:** Assistant Surgeon Buffalo General Hospital


### BUFFALO MEDICAL JOURNAL.

VOL. XL.—LVI. JUNE, 1901. NO. 11.

### Original Communications.

### THE CONSERVATIVE TREATMENT OF INJURIES OF THE EXTREMITIES.[1]

By CHAUNCEY PELTON SMITH, M.D.,

Assistant Surgeon Buffalo General Hospital.

IT GIVES me great pleasure to invite attention to a subject which has interested me greatly, and I trust may interest many of those present—namely, the conservative treatment of injuries which are usually regarded as requiring amputation or resection.

The possibilities of the subject were thrust upon me when in Baltimore. A patient came to the Hopkins Hospital, his finger cut off by some machine, carrying the injured member in a piece of brown paper and asked to have it "stuck on." I paid no attention to what I considered an unreasonable request, until one of the older residents saw the case and after carefully cleansing the stump and finger, which latter was cut off between the second and third joint, sutured the parts with silk, wrapped the wound with collodion and crepe de leisse, and told the patient to come the next day. On inquiry I found that several fingers, where the injury had been caused by edged tools, had been saved by similar means. In consequence much annoyance and regret had been caused, for the reason that patients could not understand why crushed fingers, hands and feet had to be amputated. In all, I saw about four cases treated by this method of deep suture, protection and heat. Out of the four cases one failed to unite. The ultimate results in three were good, as the patients had fair motion besides the cosmetic effect of the hand. Presumably this treatment has been done in Buffalo, but it has never been my good fortune to see it.

1. Read before the Section on Surgery, Buffalo Academy of Medicine, February 5, 1901.

I next ask your attention to *compound dislocation of the ankle with or without fracture of the fibula.* These are the cases most commonly treated by amputation or resection. Why? I never have been able to see the point of further injuring an already damaged joint by a resection, nor of removing articular surfaces whose regeneration would fall far short of a normal joint. Even modern text-books on the subject, while they mention the conservative treatment, yet give very full and explicit directions for resection. Many authors speak of drainage tubes passed through the joint, to be removed when the discharge has stopped. I should like to protest most strongly against putting drainage tubes in joints. If you, gentlemen, could but see a joint after a tube has been in a week or so. It was my good fortune to see a pyarthrosis of the knee, which was opened, drained and a half inch rubber tube was passed through laterally. The patient did not do well and the joint was opened for resection. On looking into the joint, one saw a necrotic tunnel in the cartilage of the femur where the drainage-tube had lain and killed the articular cartilage by pressure. It was a condition which I have never forgotten, and if one must drain a joint, simply keep the skin and capsule open with rubber tissue and irrigate. In compound dislocations of the ankle why should we drain? We do it more on authority or habit. As one surgeon said when I asked him in 1893 why he drained clean cases of appendectomy, he replied that he "felt better." For the past five years he no longer drains and presumably feels better yet. The surgical treatment for compound fractures by indirect violence is immediate closure. Why should it not be so for dislocations? Dislocations of the ankle in a large proportion of cases, are caused by indirect violence. Usually the violence is applied at the foot, the body being held rigid, or twisting of the foot. If, then, most of these injuries are caused by indirect violence, the wound is comparatively clean and the tissues uninjured. In all the cases I have seen the joint itself is clean, as the lower end of the tibia in bursting through the capsule and skin receives most of the dirt, and by its position protects the joint proper.

Case I.—J. J. Injured by weight falling on leg; brought to Fitch Hospital; examination revealed a compound dislocation of ankle; under chloroform wound cleansed with salt solution, lower end of tibia scrubbed with the same solution, adjacent parts shaved and cleansed, tibia slipped back in place. Wet bichloride dressing. The patient made an uninterrupted recovery.

Case II.—J. S. Patient struck by foundation stone, causing fall; was brought to the Fitch Hospital. Examination revealed a compound dislocation of ankle with fracture of the fibula, two and one-half inches above the external malleolus, protrusion of the tibia two inches from the wound, the latter being of a "Y" shape. Under chloroform, joint cavity washed with hot salt solution, lower end of the tibia scrubbed with the same solution. Wound trimmed and sutured and joint locked tight. Foot wrapped in hot salt solution and put in forced inversion. After four days, dressing removed, no local or constitutional symptoms of inflammation. Foot and leg put in side splints. Patient removed home eight days following admission. Highest temperature while in the hospital was 101° on the third day. After two weeks I saw the patient, through the courtesy of Dr. Dunham, and leg was put in plaster-of-Paris. At that time motion was possible, being one-third of normal. Observation three months after the injury, motion one-half of normal. Result, poor, if it were a Pott's fracture, but condition ascribed to patient's age, being 62, and general stiffness of the rest of the joints. Four months after injury patient complained of stiffness of joint and pain in walking. Under daily treatment by the Paquelin cautery and hot air, the joint motion improved markedly, the swelling subsided, pain almost disappeared and the motion was restored to one-half of normal.

He was not satisfied with treatment and went to the General Hospital, where he said Dr. Park removed a small piece of bone. I saw him there, but am unable to state what was done. The last observation, about nine months after the injury, patient walked lame, fair motion and could get around without the aid of a stick. Result would be classified as fair. We all have seen similar results from simple Pott's fractures.

Case III.—J. B. Foot caught in an elevator. Brought to the Fitch Hospital. Examination showed a compound dislocation of the ankle outward. Usual treatment of irrigation, cleansing. As I was associated in this case with a physician and he strongly urged drainage, I did as little damage as I could by drainage with a few strands of fine catgut. Wound dressed in four days. Normal. Plaster-of-Paris in eight days, the motion at this time being good. Position of forced inversion. Uninterrupted recovery. Slight temperature for a few days. Functional recovery excellent.

I have presented in some detail the histories of three cases, two of which have excellent functional ankle joints. In one the recovery is only fair, but I have seen the same result following Pott's fracture in the hands of surgeons with the best reputations. If the joints were resected the result, in my opinion, would not be so good, and again there would be some shortening.

I would ask your attention to two cases of pyarthrosis of the knee-joint, a condition for which a large percentage of surgeons would resect.

Case IV.—J. J. *Fractured patella by indirect violence.* Operation twenty-four hours after the injury. Capsule—not bone—sutured with gut. Uninterrupted recovery, until about the fourteenth day, when there were unmistakable signs of pus. I may say here that I have noticed this late suppuration of knee-joints a number of times, and it usually is not accompanied by much local or constitutional symptoms of suppuration. The joint was opened laterally and was washed out with salt solution; no drainage-tube; skin and capsule kept open with accordion pleated rubber tissue. This procedure was continued every other day for twelve days when the suppuration ceased and wounds healed. Patella remained firm throughout the manipulations and bony repair took place. Patient two months afterward had good union and good function of knee-joint.

What would be the present condition if resection were done?

Case V.—T. G. *Fractured patella, wiring, pyarthrosis of knee, necrosis of patella, removal of patella, profuse deep-seated phlegmonous suppuration of thigh and lower leg. Continuous immersion, recovery.* This case was an heirloom from my predecessor at the Fitch. Case first seen May 1, 1900. Was admitted three weeks previously when the wiring was done. May 5, patella became necrotic and was removed. Joint was freely exposed by anterior incision, while multiple incisions were made on the anterior, external, internal, and posterior aspect of the thigh, as the parts were the seat of profuse deep-seated suppuration. Aggregate length of incisions two feet. Notwithstanding this free drainage, the pus burrowed up the leg and patient would discharge 100-125 c.c. of pus a day. Patient chloroformed and more incisions were made below the knee, as there were evidences of pus burrowing down over and about the tibial head, but with little benefit. Patient's temperature was running 103-105 degrees, and I saw that he would die from sepsis unless radical measures were adopted. Amputation thought unnecessary and doubtful if patient would stand the shock. He was transferred to the General Hospital, where a very light dressing was put over the knee joint, but not over the incisions, and he was immersed in salt solution. A bath tub was half filled and patient placed in it, where he remained for three days and nights, after which time he was allowed from midnight until 6 a.m. in bed, after that time he was placed back in the tub. From the first the patient began to improve and after three weeks of this treatment he was placed in splints and allowed to lie in bed. Recovery slow, but after ten weeks at the hospital he was allowed to go home with a functionally good leg with the exception of a stiff knee. This condition, of course, would be expected following the removal of the patella, pyarthrosis and long continued suppuration.

It may be pertinent to enquire, the value of this procedure, and it can be summed up in the effects of heat and the mechanical effects of fluid. As fast as he made pus, it was washed out by the currents in the water, as every move would set them in motion, and the pus was absorbed by the water and not by the patient. In this case, I doubt if the salt solution was of any value as it was in a short time changed into unknown chemical compounds. But there is no question in my mind that the immersion not only saved the limb, but also the life of the patient.

Case VI.—J. S. *Lacerated wound of the index finger, infection, suppuration, threatened gangrene, immersion, recovery ; anchylosis.* Patient a machinist by occupation, hence all fingers quite important; received a lacerated wound which became infected. I treated him without much benefit for two weeks as the phlegmonous inflammation not only persisted, but spread. Incision. Dressing two days afterward and the end of the index finger down to the second joint was a dark brown color; in fact, I thought that gangrene had set in. Continuous immersion ordered and given for twelve hours. At the end of this time, I saw him and the finger which was a brownish black had changed to a rosy pink. Immersion continued. Uninterrupted recovery. Anchylosis of the distal joint.

The foregoing case illustrates the benefits of continuous immersion and its results. When the finger was on the border line of gangrene, I know of nothing except immersion which would bring it back to normal. Wet bichloride or a dry dressing would have killed it. In the majority of cases the finger would have been amputated.

Case VII.—J. S. *Compound fracture of femur, lower third,* (railroad crush). Patient admitted 3 a.m. and was seen by me a short time afterward. Examination showed a compound comminuted fracture of the femur with extensive laceration of tissue down to the bone. A flat car was said to have passed over the leg. I suggested amputation, but the patient was unwilling until his friends could be communicated with. Saw patient at 9.30 a.m. the following day and on closer examination found his femoral artery uninjured. How it escaped I do not pretend to say. An idea occurred to me following out a writer in the Annals of Surgery, who in a case of mangle injury crushing the radius, resected the ulna to an equal extent of bone lost in the radius; the ends were approximated, which made a "U" shaped mass of the tissues on the ulnar side of the arm, which carried the vessels. Several months afterward when union had taken place, he cut this "U" off, leaving a functionally useful arm with flexion and extension of the fingers, the arm being shortened by three inches. With this procedure in mind, I cut away all the extensor, abductor and adductor muscles down to the bone and trimmed away all the tissue necrotic by pressure. The femur was resected for three inches and the ends approximated and sutured with heavy silver wire. When the ends of the bones were approximated the muscles needed to be trimmed, so as to get better union and when this was finished the leg had a diameter of the midthigh. The femoral artery was exposed for a distance of three inches, and I was careful not to twist or compress it when approximating the soft tissues. Wound closed without drainage. Patient recovered from chloroform, talked to his friends and with me. Was in excellent condition until 7 p.m. when he suddenly died. Cause unknown.

I was satisfied from the work done that the man would eventually have a functionally good leg with knee and ankle joint motion. Hereafter I will not amputate in similar cases, as I believe while this case had an unfortunate termination, yet the principle is worth trying for.

Case VIII.—*Compound comminuted fracture of the bones of the carpus, lacerated wound of the wrist, compound fracture of ulna, suturing wound, wiring bone, immersion, recovery.* Patient was bitten by a bear, receiving a lacerated wound of the wrist and a compound comminuted fracture of two bones of the carpus, ulnar side. Joint laid open and hand disarticulated. Hand was held to forearm by the extensors of the thumb (ext. ossis metacarpi pollicis, ext. primi internodii pollicis, ext. secondi internodii pollicis) blood supply, the radial artery. Patient also had a compound fracture of the ulna, which does not concern us, except as a complication, and which may be disposed of by saying that the bone was wired; union perfect; wire still in. Patient had been seen by two or more physicians who said that the hand would have to come off. Saw patient two hours after the injury and was inclined to think that amputation would be necessary owing to the extent of the laceration of the wrist. On examination, radial artery was found to be intact, so after explaining to the patient that failure might result, the arm was treated conservatively. Small fragments of bone were removed, tendons trimmed and joined, hand sewed back in position, and wound closed without drainage. Continuous immersion for seventeen days. Patient was seen twenty-four hours after the injury and the condition was excellent; hands warm, finger nails rosy. Patient was left in the care of Dr. Gerber, who followed the conservative treatment and took most excellent care of the man. Recovery slow, owing to long-continued suppuration about the dorsum of the hand and wrist, which burrowed in several directions. The abscess was freely opened and finally all sinuses were healed. Anchylosis was broken up several times under chloroform, but without much permanent benefit with the exception of improvement of motion in fingers. Patient discharged four months after injury.

Present condition. Practically complete anchylosis of wrist. Abduction and adduction of the thumb and fingers one half of normal, which permits patient to grasp and hold objects between thumb and forefinger. Flexion and extension of fingers about fifteen degrees. Hand, as a whole, one-tenth of normal usefulness, but, in my judgment, far better than an artificial member aside from the cosmetic and mental effect of possession.

In conclusion, I wish to ask a trial for the conservative treatment of the class of injuries, to which I have alluded, and also to thank you for your attention this evening.

90 N. PEARL ST.

